# Nutritional Value of *Colocasia esculenta* Is Related to Corm Size

**DOI:** 10.3390/life15111712

**Published:** 2025-11-05

**Authors:** Albert Thembinkosi Modi

**Affiliations:** Department of Biological and Environmental Sciences, Faculty of Natural Sciences, Walter Sisulu University, Mthatha 5099, South Africa; amodi@wsu.ac.za; Tel.: +27-(0)722074325

**Keywords:** corm size, macronutrients, micronutrients, fibre, yield

## Abstract

Taro (*Colocasia esculenta*) is a tropical root crop widely cultivated for its edible corms and leaves. The objective of this study was to determine the effect of taro morphometric parameters on prolificacy, yield and nutritional value under dryland production. Two sites were used to grow small, medium and large propagules generated under controlled environment conditions from a local landrace. Plant prolificacy, in terms of corms per plant, crop yield (t·ha^−1^) and nutrient content (macro- and micronutrients) and fibre were used to determine taro quality. The size of propagule was associated with both productivity and nutritional value. There was a positive correlation between propagule size and starch content. A decline in both Acid Detergent Fibre (13%) and Neutral Detergent Fibre (25%) occurred in larger corms. The protein and macronutrient contents improved with corm size, but the micronutrient content decreased. This study revealed that there are benefits in the utilisation of a wide range of corm sizes for upland production purposes. However, there is a need to investigate and expand knowledge of taro food components to include its potential value for specific nutritional and industrial purposes.

## 1. Introduction

Taro (*Colocasia esculenta*) is a tropical root crop widely cultivated for its edible corms and leaves [1,2]. The crop thrives in warm, humid environments with temperatures between 21 and 27 °C. It requires consistent moisture, ideally 1500–2000 mm of annual rainfall. It prefers deep, fertile, well-drained loamy soils with a pH of 5.5–6.5. Typically, taro is propagated vegetatively using corms (main underground storage organ), cormels (small daughter corms) or suckers (shoots from the base of the plant). Corms are best for high-yield commercial production due to their vigour and fast establishment, but they are bulkier and more prone to disease. Cormels are easier to handle and store, suitable for small-scale farming or when planting material is limited. Suckers are ideal for rapid multiplication and nursery propagation, especially in controlled environments. Despite the dominance of rice, wheat, maize and potato as global staple food crops, taro is one of the traditional staple crops of Southeast Asia, the Pacific and West Africa. Genetic exploitation of the crop in the areas of its origin and traditional use is limited. This has led to a limited knowledge about the agronomy of taro varieties and cultivars. Where progress has been made regarding taxonomy and ethnobotany, more efforts are required to expand agronomic and food science aspects regarding the role of taro in food security. As taro cultivation increases beyond the traditional tropical zones, new producers need a scientific basis for advice on the selection of taro propagules for yield and nutritional value of the harvested crop.

Propagule morphology is a heritable genetic trait [3]. Purposeful selection of vegetative seed and propagule size to influence crop yield has been widely adopted in agriculture as part of crop improvement strategies, which include science and technology [4,5,6,7]. For example, under appropriate crop management conditions, large seed potatoes are generally associated with vigorous plant growth and high yield. Propagule quality for taro production influences crop establishment, growth and yield [8]. Previous research has also shown that propagule size has a direct positive relationship with crop yield due to higher stored energy and nutrient content. However, seeding rate has a more significant effect on crop yield because of its close relationship with photosynthetic capacity and net assimilation rate [9]. Although taro is well known for its good nutritional value [10], previous research has focussed more on environmental effects and crop management for yield and less on nutrient content in relation to planting material (propagules) [11].

Previous research indicated that nutritional comparison of taro, potatoes, and sweet potatoes, based on typical values per 100 g of cooked root, reveals interesting trends [12,13]. Taro is higher in carbohydrates and fibre than both potatoes and sweet potatoes, making it more filling and better for digestive health. Potatoes have more vitamin C and slightly more protein, but a higher glycemic index (GI). Sweet potatoes are rich in vitamin A (beta-carotene), making them excellent for eye health and immunity. Selecting planting material for taro can be based on crop production goals or environmental conditions. Environmental conditions play a critical role in the successful production of root and tuber crops such as potatoes, sweet potatoes, cassava, yams and taro. These crops are sensitive to various factors that influence their growth, yield and quality. In a detailed review of the relationship between crop quality and yield of root and tuber crops, it was stated that “strategies that are included in the category of adaptation tactics are agronomic practices, crop diversification, improved water management, breeding for climate resilience, and agroecological techniques” [14]. However, the general focus of research disregards the need for a multidimensional view of crop quality and nutrition in response to management practices. Sustainable agriculture requires an understanding of a balanced approach regarding the environment and nutrition [14]. Soil type, temperature, moisture levels and sunlight exposure significantly influence the growth, yield and quality of root and tuber crops. Understanding these factors can lead to improved cultivation practices and food security [15]. The hypothesis of this study was that a taro landrace propagule affects yield, but it may not affect the nutritional content of the harvested corms. The aim of this study was to determine the relationship between the morphometric parameters of taro propagules with corm size per plant (prolificacy), yield (productivity) and nutritional content under upland field conditions.

## 2. Materials and Methods

**Figure 1:**
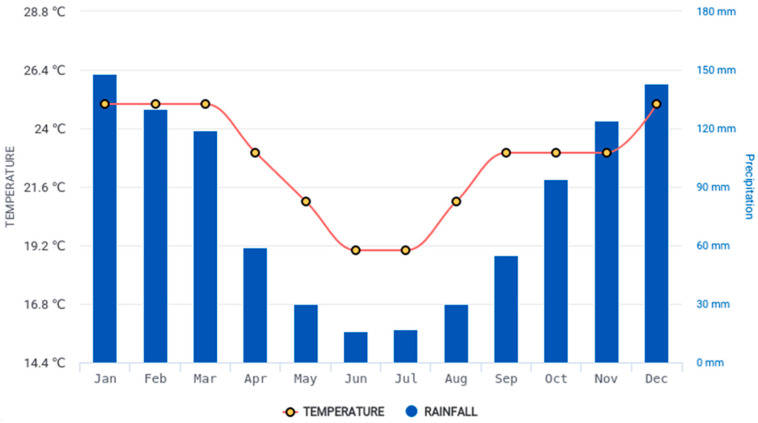
Climatic data.

This study was conducted under dryland conditions (upland) on a research farm in Pietermaritzburg (−29°37′0.44″ S 30°23′34.01″ E), South Africa, during the 2022/2023 growing season, with average climatic conditions reported by the South African Weather Service (Figure 1) [16]. Soil samples were randomly taken from two sites at the location to represent two experimental blocks for data collection. The results of the soil analysis based on the standard *Soil Analysis Handbook of Reference Methods* (https://www.taylorfrancis.com/books/mono/10.1201/9780203739433/soil-analysis-handbook-reference-methods-soil-plant-analysis-council-inc (accessed on 25 September 2025)) are shown in Table 1.

**Figure 2:**
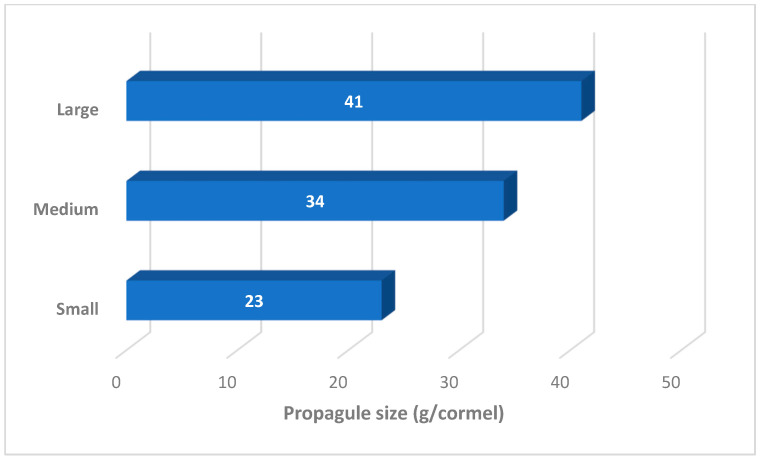
Morphometric differences of taro planting material were indicated by mass. Significant differences (*p* ≤ 0.05) were determined using an analysis of variance (ANOVA) and least significant difference (LSD) test (1.4).

**Figure 3:**
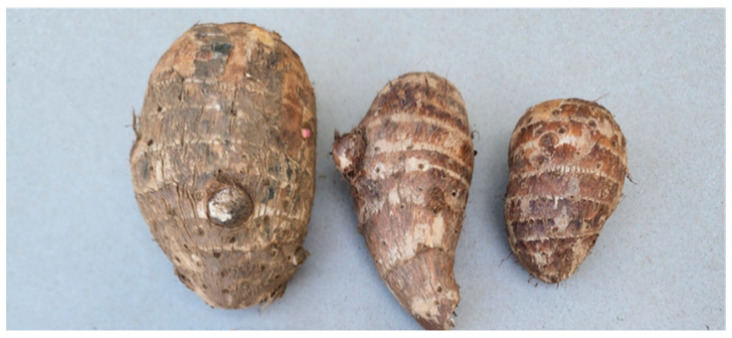
Visual presentation of taro propagules used for planting, designated by size: large (**left**), medium (**middle**) and small (**right**).

This study used landrace material of Dasheen-type taro identified as Umbu1 [17]. Planting corms were originally collected from 100 farmers in Umbumbulu (29.9889 S, 30.7003 E), South Africa, more than 60 km from the study location (Pietermaritzburg), where the germplasm was preserved in situ for propagule preparation. Propagules were separated based on morphometric parameters [18]. Morphometric measurements of taro corms include key dimensions typically assessed in agricultural and botanical studies. These are corm length (from top to bottom), corm diameter (widest horizontal section), corm weight (usually measured separately) and shape characteristics (e.g., cylindrical, conical or irregular). Corm uniformity variation is expected for landrace germplasm [15] (Figure 2 and Figure 3). The planting material was prepared under controlled environment conditions as previously documented and planted using the organic method [16,17]. Harvesting occurred at physiological maturity, indicated by full leaf senescence, six months after planting. Chemical analysis was performed in the raw taro corms, after they were processed into powder using a modification of previously published methods [19,20].

A factorial experiment was used (Table 2). Location (site) was treated as a block and corm size as the main plot. Within each block, corms were planted at the depth of 20 cm in 4 m^−2^ plots to establish a plant population of 45,000 plants per hectare, as prescribed for upland production [18]. The sub-plots for sampling were the two middle rows of the main plot to create border rows. The experiment was a completely randomised block design, replicated four times (Table 2). An analysis of variance (ANOVA) was used to determine differences between planted corm sizes (treatments) with respect four variables, namely, the size of harvested corms, number of corms per plant (or planted corm), yield and nutritional content of the harvested corms. Differences between mean values were determined using least significant difference (LSD) (*p* ≤ 0.05).

## 3. Results

**Figure 4:**
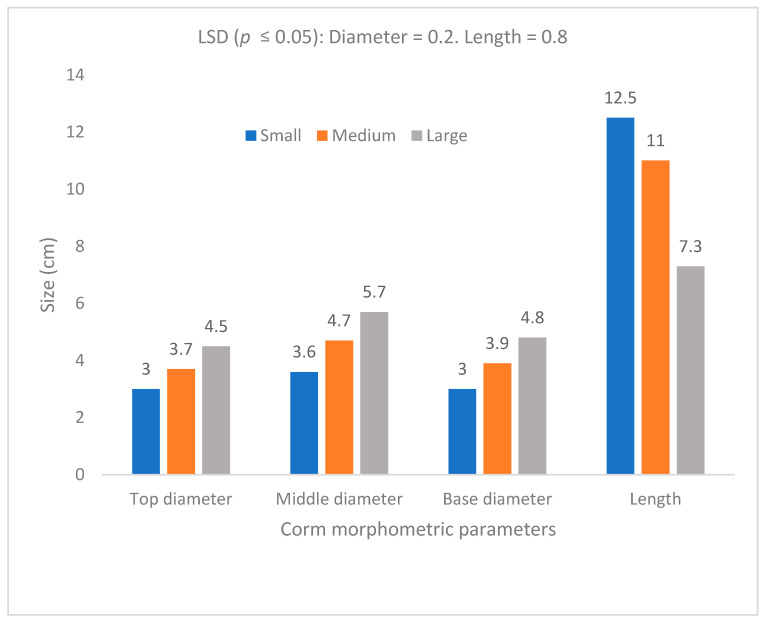

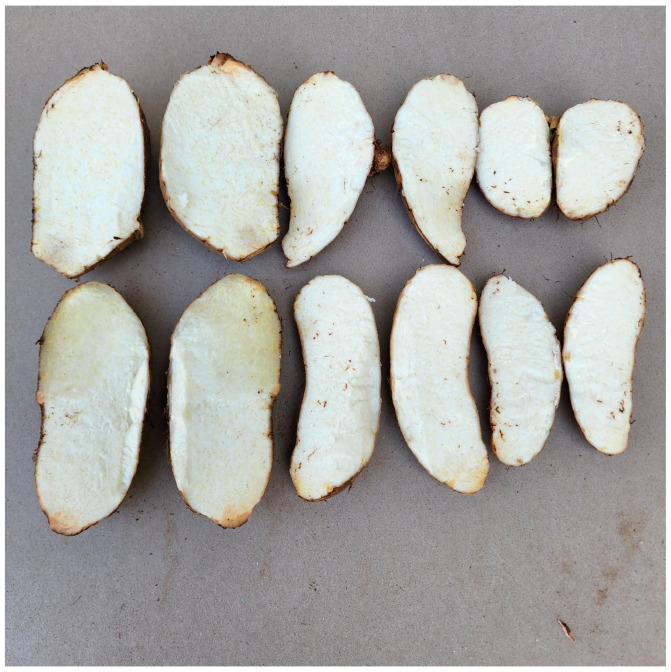
Corm morphometric measurements (**top**) and illustration (**bottom**). The visual illustration shows large, medium and small taro corms (from left to right).

**Figure 5:**
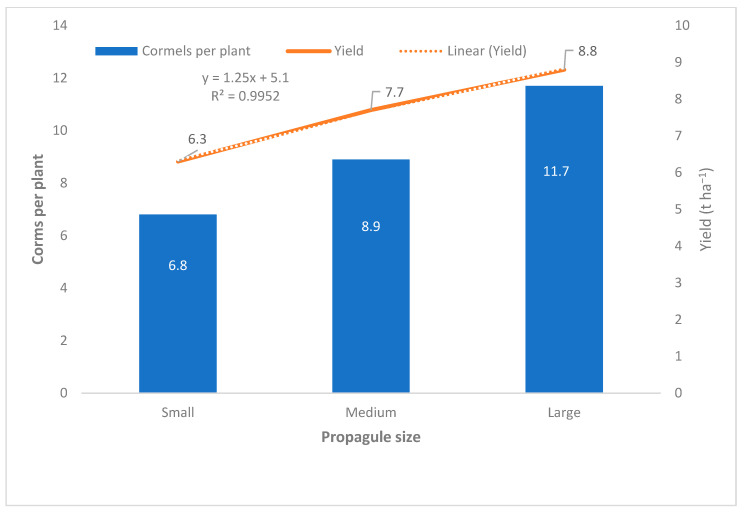
Corm production in relation to propagule size. ANOVA LSD (*p* ≤ 0.05).

The propagules were cut to determine size in terms of length and diameter (cm). This study found no significant differences between sites. The results showed that there is a significant difference (*p* ≤ 0.05) between corm sizes (large, medium, small) with respect to length and diameter morphometrics, irrespective of corm position (Figure 4). The corm was widest in the middle section (4.7 cm). The base and top sections were 20% and 15% narrower than the middle section, respectively, but there was no significant difference between these two sections (Figure 4). The harvested corms were directly correlated to propagules in terms of the number of corms per plant (prolificacy) and yield (Figure 5). The large propagules produced 42% and 24% greater yield than the small- and medium-sized propagules, respectively (Figure 5).

The number of harvested corms per plant was used as an indicator of prolificacy (Figure 5). It was important to determine the relationship between planting materials (propagules), prolificacy and corm yield. This study showed a significantly positive relationship (*p* ≤ 0.05) (Figure 5).

**Figure 6:**
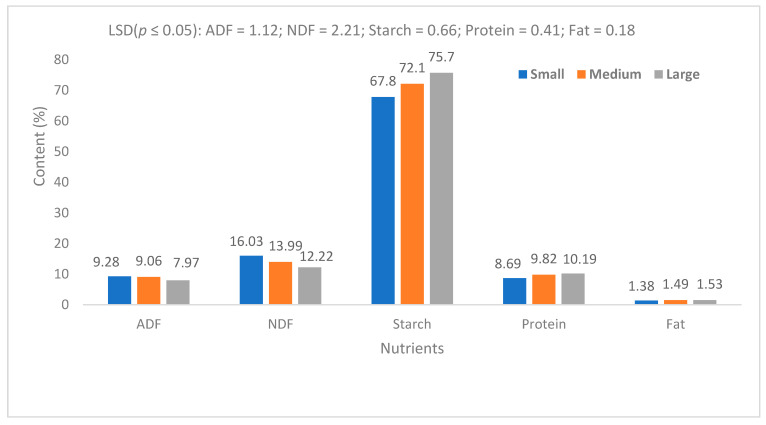
Nutrient content of harvested taro corms (dry weight). ADF = Acid Detergent Fibre. NDF = Neutral Detergent Fibre.

**Figure 7:**
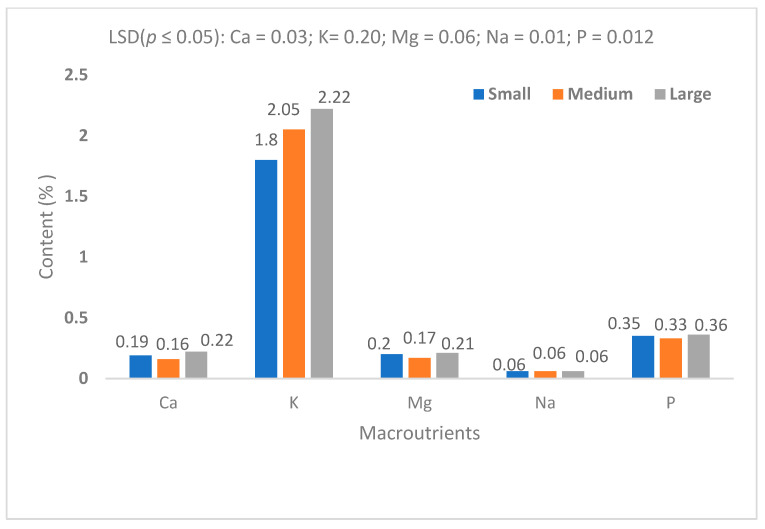
Macronutrient contents of harvested taro corms (dry-weight basis).

**Figure 8:**
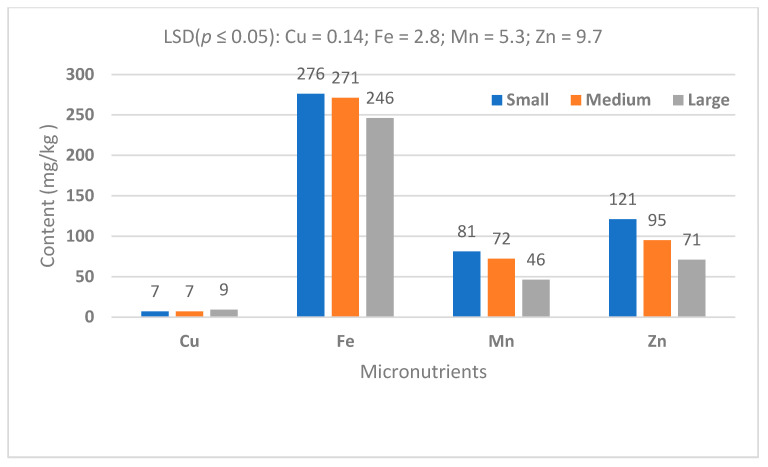
Micronutrient content of harvested taro corms (dry-weight basis).

Taro corm size was associated with nutrient composition, namely macro- and micronutrients, respectively (Figure 6, Figure 7 and Figure 8). Fiber content is an important indicator in staple food and forage crops. This study showed a significant decrease (*p* ≤ 0.05) in Acid Detergent Fibre (ADF) as well as Neutral Detergent Fibre (NDF) in corms derived from large propagules compared with those derived from small propagules (Figure 6). However, there were no significant differences between small- and medium-sized propagules with respect to both ADF and NDF (Figure 6). The starch content consistently improved as the propagule size increased (Figure 6). The large propagules showed significantly (*p* ≤ 0.05) higher protein content than the small propagules, but there was no significant difference between the large and medium propagules (Figure 6). Propagule size had no significant effect on the fat content of harvested corms (Figure 6).

In plant tissue analysis, macronutrients are typically present at concentrations ranging from 0.1% to 5% of dry weight, reflecting their roles in structural and metabolic functions. In contrast, micronutrients are required in much smaller quantities and are usually found at concentrations between 5 and >100 mg/kg of dry weight. This distinction in concentration and unit of measurement underscores the differing physiological roles and uptake requirements of macro- and micronutrients in plant nutrition (Figure 8). In this study, potassium (K) and iron (Fe) were the predominant essential minerals found in taro corms, irrespective of propagule size (Figure 7 and Figure 8). Sodium (Na) was the macronutrient found in the lowest concentrations, followed by Mg and Ca when corms produced from all propagule sizes were compared. The copper (Cu) concentration was found to be the lowest among micronutrients compared with Mn, Zn and Fe (Figure 8).

## 4. Discussion

This study focused on propagule size as a morphometric parameter of one taro genotype. Crop performance and yield potential are related to genetic effects. However, environmental conditions and management conditions may have a more significant effect. The quality of propagules, which are the planting material used for taro, is important for stand establishment. This ensures early crop development and efficient utilisation of resources. However, it is important to note that the Leaf Area Index (LAI) and Photosynthetically Active Radiation (PAR) are closely linked in crop production, but LAI does not enhance PAR directly. Instead, it influences how effectively PAR is intercepted and utilized by the crop canopy. Water use efficiency is improved by early crop development, leading to physiological maturity. This phenomenon may be associated with higher energy and nutrient reserves in large propagules compared with small ones to support seedling growth. Climate change is increasingly making it difficult to produce a uniform crop under rainfed conditions. Therefore, producers do not always have the option of maintaining uniform propagule quality for crop production. Root and tuber crops have been generally assumed to have similar characteristics with respect to the size of planting material. For example, the size of seed potatoes plays a crucial role in determining crop yield, and its scientific relevance is well documented in agronomy and horticultural research [20,21,22,23]. Larger seed potatoes contain more stored carbohydrates and nutrients [24]. These reserves support early sprout vigour, root development and shoot growth, especially under suboptimal conditions. Larger tubers often produce more sprouts, which can lead to more stems per plant. This can increase the number of tubers formed, although it may also lead to competition among stems if not managed properly. Uniform and vigorous emergence from larger seed pieces contributes to better canopy development, which enhances photosynthesis and tuber bulking [25]. Previous studies showed that medium-sized seed potatoes (typically 50–80 g) often yield better. Very small seed pieces may result in weak plants, while very large ones may be uneconomical and prone to disease. Field trials often compare whole vs. cut seed potatoes and different size classes (e.g., <30 g, 30–50 g, 50–80 g, >80 g). However, the results also vary with variety, soil type, climate and management practices. Medium-sized seed pieces generally offer the best performance in terms of yield per hectare. These findings were shown for taro in the current study.

This study confirmed that propagule size can be consistently associated with crop performance in terms of corm prolificacy and yield. However, the relationship between propagule size and nutrient content cannot be generalised in terms of corm size. There are consistent comparisons with respect to the concentration of various nutrients. The major finding of this study is that in addition to higher starch content, taro corms produce significantly high levels of protein and fibre. Propagule size is important in determining macro- and micronutrient variations in taro corms. Taro starch is composed of complex carbohydrates, and this contributes to slow release of dietary energy compared with the major staple crops. The high fibre content is beneficial for regulating a slow absorption rate of sugar into the bloodstream [26]. Although the protein content is low, it is significant in terms of taro contribution to its overall nutritional profile.

**Figure 9:**
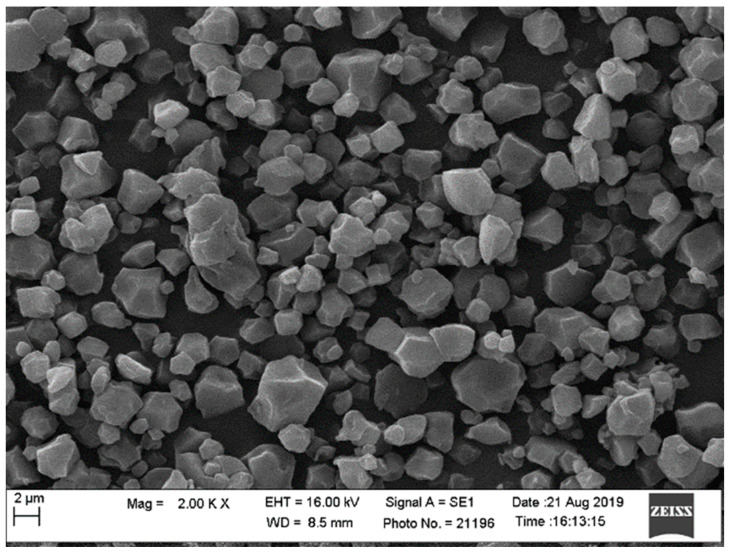
Scanning electron microscopy visualisation of taro starch grains [22].

Previous studies have also shown that the nutrient content of taro is directly associated with the soil nutrient profile [15]. In this study, soil profile could not be directly linked to taro nutrient content. For upland production, water availability is more important for nutrient absorption by the plant. Temperature, humidity and sunlight exposure can influence the growth rate and nutrient accumulation. It has been shown that variation in taro nutrient content is highly associated with variety, in that some varieties are naturally richer in certain nutrients than others [19]. This study showed that under dryland conditions, taro nutrient content is influenced by propagule size. Large propagules produce higher levels of potassium compared to smaller ones. Iron, magnesium and zinc concentrations are higher in smaller propagule corms compared with large ones. It is significant to note that while the findings confirmed the well-known value of taro as a high-energy crop in terms of starch content, the new knowledge can be associated with the relationship between corm size and fibre type and content. This is important from both human food and animal feed perspectives, in that fibre content influences digestibility and absorption of energy [17]. High crude fibre indicates low digestibility. Acid Detergent Fibre includes cellulose and lignin, which are less digestible. Neutral Detergent Fibre includes hemicellulose, cellulose and lignin, providing a more accurate plant fibre content for nutrition. In this study, large corms contained approximately 13% less ADF compared with small corms. Large corms contained approximately 25% less NDF compared with small corms. Previous research [19] showed that taro starch grains are uniform (Figure 9), but the results of the current study suggest that there may be a possible variation linked to nutrient quantity and corm size. This explanation requires further investigation in the context of utilisation of taro crop as a food source and for further investigation of its potential pharmaceutical value [25,27,28].

Root and tuber crops such as cassava (*Manihot esculenta*), sweet potato (*Ipomoea batatas*), yam (*Dioscorea* spp.) and taro (*Colocasia esculenta*) are staple foods in many tropical and subtropical regions, valued for their high starch content, dietary fibre and essential minerals. The nutritional composition of these crops is significantly influenced by soil quality, which encompasses physical structure, chemical properties and biological activity. Soil pH, nutrient availability, and microbial dynamics directly affect the accumulation of starch and the bioavailability of minerals such as iron (Fe), zinc (Zn) and copper (Cu). For instance, acidic soils may enhance the solubility of micronutrients but can also lead to toxicity or reduced microbial efficiency. Conversely, alkaline soils often limit micronutrient uptake, thereby diminishing crop nutritional value. Optimal soil pH for root and tuber crops typically ranges between 5.5 and 6.8.

Dietary fibre content in these crops varies widely, with some species contributing significantly to the daily recommended intake. Mineral bioavailability also differs, with Zn and Cu showing relatively high absorption rates, while Fe remains poorly bioavailable, posing challenges to addressing micronutrient deficiencies through these crops alone. The implications for crop quality are profound. Poor soil conditions can lead to reduced starch synthesis, lower mineral concentrations and compromised fibre levels, ultimately affecting both yield and nutritional value. Integrated soil fertility management, including organic amendments and crop rotation, alongside the selection of genetically improved cultivars, is essential for enhancing crop quality and resilience. This study provides information about the value of taro landrace germplasm.

## 5. Conclusions

Successful agriculture depends on a combination of plant genotype, soil environment, climate and management factors. The focus of this study was on the relationship between taro morphometric parameters and crop performance with respect to yield and nutritional value. The findings should be considered in the context of the upland agroecosystem for taro production, which may not be comparable with wetland agroecosystems. Upland or dryland genotypes are characterised by greater variability of corm size, which makes crop quantity and quality less predictable. Therefore, the contribution of this study to new knowledge is the explanation of the role of phenotype morphometric parameters in taro crop production under dryland conditions. The major findings relate to the significant effect of propagule size on both the yield and nutritional value of the harvested crop. The findings suggest that taro corm size enhances crop performance in terms of prolificacy (corms per plant) and total harvestable yield. This may be due to the starch content and energy value. Neutral Detergent Fibre and protein contents are improved by high-quality propagules, which produce more and larger corms. The potassium content improves with corm size, but the Fe, Mn and Zn contents decrease. The limitation of this study lies in the lack of crop development information from emergence to harvest maturity, which could explain crop physiological quality with respect to the optimisation of photosynthetically active radiation, water use efficiency and harvest index, among others. Future studies would benefit from a combination of agronomy, physiology and biochemistry aspects of taro corm size effects under a wide range of genotypes and agroecosystems. Scanning electron microscopy would assist in confirming whether corm size and climatic conditions influence electromagnetic features such as starch grain size and composition. This information may be useful for industrial and pharmaceutical purposes. It is recommended that producers use large corms for planting due to their high energy content. However, for purposes of nutritional value, large corms are favourable carbon-based nutrients, while smaller ones contain higher levels of micronutrients. These are important considerations for distinguishing taro use for food security and pharmaceutical purposes.

## Figures and Tables

**Table 1:** Soil analysis results for the two study sites (Site 1 and Site 2).

	Units	Site 1	Site 2
Density	G·mL^−1^	0.8	0.82
P	mg·L^−1^	12	15
K	mg·L^−1^	100	106
Ca	mg·L^−1^	317	314
Mg	mg·L^−1^	130	130
Exchangeable acidity	cmol_c_L^−1^	2.91	2.15
Total cations	cmol_c_L^−1^	5.82	5.06
Acid saturation	(%)	50	43
pH	(KCl)	4	4.08
Zn	mg·L^−1^	2.1	2.3
Mn	mg·L^−1^	3	4
Cu	mg·L^−1^	2.5	2.6
Organic carbon	%	4.6	4.2
N	%	0.28	0.23
Clay	%	55	51

**Table 2:** Experimental design for analysis of variance (ANOVA).

Design type (factorial in RCBD)	Blocking Factor: Field variability (field treated as a block) Main Plot Factor: Corm size (small, medium, large) Subplots: The two middle rows of each main plot are used for sampling.
Sampling method	Sampling Unit: Plants within the two central rows of each main plot.
Sampling technique	Random sampling within the central rows. 5 plants per subplot for uniformity. Consistent spacing between sampled plants to avoid bias.
Statistical analysis	ANOVA for factorial RCBD: Main effects: Corm size Interaction effects (if a second factor is included) Replication: Number of blocks = number of replications Error terms: Main plot error (between corm sizes) Subplot error (within corm sizes)

## Data Availability

No new data were created or analyzed in this study. Data sharing is not applicable to this article.
